# Checklist of edible insects and their marketability: improving rural livelihood in Imo State, Nigeria via insect consumption and marketing

**DOI:** 10.3389/finsc.2026.1856335

**Published:** 2026-08-07

**Authors:** John I. Iwunze, Bertram E. B. Nwoke, Micheal O. Nwachukwu, Venatius C. S. Ubah, Gerald M. Ugagu, Nnedinma N. Nnodim, Murphy C. Nwoke, Ozioma P. Ugwuegbulam, Chizoba Chidera Amaechi, Onyinyechi C. Ndubueze, Samuel M. Dadi, Kelechi K. Okwara, Gospel U. Nzewuihe, Udoaku C. Egbe, Williams Nongu, Onyinyechi G. John, Goodluck E. Alisi

**Affiliations:** 1 Department of Biology, Federal University of Technology, Owerri, Imo State, Nigeria; 2 Department of Animal and Environmental Biology, Imo State University, Owerri, Imo State, Nigeria; 3 Department of Biology, University of Innovation, Science and Technology, Omuma, Nigeria; 4 Department of Biology, Alex Ekwueme Federal University, Ndufu-Alike, Ebonyi State, Nigeria; 5 Department of Biology, Alvan Ikoku Federal University of Education, Owerri, Imo State, Nigeria; 6 Department of Animal and Environmental Biology, Kingsley Ozumba Mbadiwe University, Ogboko, Imo State, Nigeria

**Keywords:** constraints, edible insects, Imo State, marketability, rural livelihood

## Abstract

**Introduction:**

The consumption of edible insects (EIs) as food has been a long tradition for some populations worldwide. They are sold in markets found in the rural and urban areas of Imo State, Nigeria. Data on the economic value, marketing, and utilization of EIs are limited, particularly in Southeast Nigeria where the study was conducted. This study is necessary for the provision of data on effective marketing and utilization of EIs in order to determine their sustainability in relation to the current market system.

**Methods:**

This study investigated the marketability of some EIs as a means of improving rural livelihood in Imo State, Nigeria. Data were collected using structured and validated questionnaires administered interpersonally to 60 respondents/EI marketers in the selected markets. Descriptive and inferential methods were employed in data analysis.

**Results:**

The study revealed that four species of EIs were sold: *Macrotermes* species (35.0%), *Gonimbrasia belina* (30.0%), *Rhynchophorus* species (25.0%), and *Anaphae* species (10.0%), with more than one species sold by marketers (43.8%). EIs were primarily obtained from the wild (68.3%) and were sold mainly for consumption (91.7%); marketing is dominated by women (91.7%). The supply of insects is irregular and seasonality is a major constraint (51.7%). *Macrotermes* species (termites) had the highest significant mean quality per marketer (*p* < 0.05). There was a significant difference in mean quality per marketer of *G. belina*, *Rhynchophorus*, and *Anaphae* species (*p* < 0.05).

**Discussion:**

The study concluded that EI marketing is a promising venture that needs attention. Policy intervention that will facilitate mass production/domestication of EIs and modern technical skills on harvesting is recommended.

## Introduction

Based on statistics from 2019, the average daily per capita consumption of protein (45.4 g) in Nigeria is less than the recommended minimum daily per capita consumption of protein by the Food and Agricultural Organization (FAO) (53.8 g), which is also below the average daily global intake (64 g). This reveals that there exists protein insufficiency in Nigeria (1–3). The availability of conventional and regular sources of animal protein like beef, pork, fish, and poultry is decreasing due to high feed cost, ancient animal husbandry, techniques, diseases, and low productivity (4). Similarly, insects have historically been a key, protein-rich food source for many rural African communities (5–7). The consumption of animal protein in Nigeria is 5.5 g per feed per day and falls below the FAO recommendation of 35 g per head per day (8). Edible insects (EIs) drive socioeconomic growth, boosting incomes and alleviating poverty for rural populations (9–11). A call has been made to harness, accept, and develop local traditional and natural foods as replacement to junk and adulterated food items, namely, foreign foods. The advantages of consuming these include naturality, nutritional relevance, longevity, ease in preparation, low cost, safety, availability, and variety (12). Global data suggest that over 2,000 insect species are deemed edible (13, 14), with more than 2 billion people incorporating them into their regular diets (15, 16). Nevertheless, the consumption of EIs has yet to gain widespread acceptance among Western consumers (17–19). Various non-conventional animal protein sources like entomophagous/EIs, crickets, winged termites, palm weevils, and grubs are now being explored. Entomophagy, or the consumption of insects, plays a significant role in the dietary habits of certain countries, offering substantial nutritional and health benefits (20). Entomophagy constitutes a traditional culinary practice, particularly prevalent within Africa, Asia, and Latin America (21). Though EIs are mainly gathered from the forests, efforts on domestication have been intensified. Insects are tipped worldwide as a recognized food. However, in Africa, insect eating is part of our menu for centuries (22). EIs serve as food for people and as a source of income. Owing to their dense nutritional profile, including essential proteins, vitamins, and minerals, they serve as an ideal alternative to conventional protein sources (23). EIs are sold in markets found in the rural and urban areas of Imo State, Nigeria. They are mainly harvested during seasons of availability, scores of species are prominent items of commerce, and the income generated could help families achieve economic independence. EI marketing is a seasonal business in most parts of West Africa and has the potential to create jobs along its value chain. EI markets are traditional, crude, and small with untapped potential. Data on the economic value, marketing, and utilization of EIs are limited, particularly in Southeast Nigeria where the study was conducted. This study is necessary for the provision of data on effective marketing and utilization of EIs in order to determine its sustainability in relation to the current market system.

## Materials and methods

This study was conducted in Imo State, which is located between latitude 4^0^45^1^N–7^0^15^1^N and longitude 6^0^50^1^E–7^0^25^1^E. The state is inhabited by Ibos and has a tropical climate that supports rainforest vegetation. The inhabitants are mainly farmers. A checklist was used to survey the marketability of EIs. Three local government areas (LGAs), one from each geopolitical zone, were randomly selected in addition to Owerri Municipal that was purposively chosen. In each LGA, three major markets were selected (except Owerri Municipal, which has only one market, EKEUKWU) and visited at three different times (on three different market days). In each of these markets, six respondents/marketers were randomly selected and interviewed (details are in **Table 1**). The aim of this market survey was to identify EI species offered for sale, unit of measurements, cost per unit, and quantities offered for sale in each market.

**Table 1:** Sampling procedures of the study area.

LGAs (locations)	Markets	No. of respondents (EI marketers)
Mbaitolu	Orie Ogbaku	6
(Lat. 5583110N)	Eke Orogwe	6
(Long. 6963290E)	Afor Irete	6
Obowo	Orie Alike	6
(Lat. 5568326N)	Eke Avutu	6
(Long. 7400972E)	Ekeja Ehume	6
Orlu	Orie Umuna	6
(Lat. 585278N)	Orie Okporo	6
(Long. 7016759E)	Eke Eziachi	6
Owerri municipal	Eke Ukwu	6
(Lat. 5476310N)		
(Long. 7025853E)		

### Data analysis

Descriptive statistics and one-way analysis of variance (ANOVA) were used to analyze the variables to determine statistical significance, with the *p*-value set at 0.05.

## Results and discussion

The results herein indicated that EIs are important items of commerce in the study areas as have been reported elsewhere (22, 24) and contribute to the socioeconomic wellbeing of the inhabitants. Their display for sale on tables and showcases places them in competition with meat products while their abundance points to quantities that are enough to meet household demands and sale. EIs no doubt play a vital role in poverty alleviation and the protein need of the people (22).

### Socioeconomic characteristics of EI marketers and respondents

Socioeconomic characteristics (**Table 2**) revealed that most of the respondents were within the age group 26–40 years (38.3%) representing economically active age group participants (22, 24–27). Soulley and Sanni (28) had reported that women not only are consulted but also play a vital role in the marketing of livestock and other related activities. Majority (91.7%) of the respondents were women and only 8.3% were men. This indicated that women dominated the market and are more involved in marketing activities than men. Results further projected that majority of the marketers were married (78.3%), while 16.7% and 5.0% were single and divorced, respectively. This points to the fact that all categories of people were involved in EI markets though they are dominated by married women (29). These married women are responsible and are into this business to supplement household income. The trade did not require a high level of education, which could be due to the primitive nature of the trade or poor fund outlay. All the EI marketers claimed that their products are seasonal, and among them, 35% had 5–6 years of experience, 31.9% had more than 6 years of experience, and 21.7% claimed having 3–4 years of experience, indicating full awareness of the market. Approximately 42% of the EI marketers sell their products in the local markets while 31.7% and 23.3% sell theirs at urban and roadsides, respectively. This showed that there is availability of market outlets in the area. The marketers had no union controlling their affairs. Thus, there is a need for authority in charge of food and related activities like consumer protection agencies and market authority to oversee/regulate their activities. Nearly half (48.3%) were engaged in marketing both termites and other EIs, with the majority obtained from the wild and 95% joined the trade by inheritance; 61.7% were involved in wholesale, while 38.3% were involved in wholesale and retail combined.

**Table 2:** Socioeconomic characteristics of EI marketers/respondents in the study area.

Variables	Frequency	Percentage (%)
Age group		
<25	9	15.0
26–40	23	38.3
41–55	19	31.7
>55	9	15.0
Sex		
Male	5	8.3
Female	55	91.7
Marital status		
Married	47	78.3
Divorced	3	4.0
Single	10	16.7
Education level		
Primary	19	31.7
Secondary	20	33.3
Tertiary	0	0.0
No formal education	21	35.0
Supply of products		
Regular	0	0.0
Not regular	60	100.0
Years of experience		
1–2	6	10.0
3–4	13	21.7
5–6	31	35.0
>6	19	31.7
Marketing channels		
Local/domestic	25	41.7
Urban	21	31.7
Road side	14	23.3
Membership of association		
Yes	0	0.0
No	60	100.0
Types of EIs sold		
Termites only	21	31.7
Termites and others	29	48.3
Palm weevil only	6	10.0
Palm grub only	3	5.0
Methods of EI acquisition		
Wild	53	88.3
Other sources	7	11.7
Form of EI marketing		
Wholesale only	37	61.7
Wholesale and retail	23l	38.3
Knowledge of EI marketing		
Inheritance	57	95.0
Training	3	5.0

### Management practices of EIs

Management practices (**Table 3**) revealed that 35% of the termites, 30% of palm weevils, 25% of palm grub, and 10% of lepidopteran larva (ololol) were found in markets. Increased awareness of nutritional benefits could be the reason (22). Termites were the predominant species due to their high acceptability, seasonal availability, and quantity. Thus, they add more revenue to the marketers. Forty-five percent stored EIs (for a day to weeks) in drums with slices of pepper after frying them to ward off microbes. However, 40% do not preserve EIs, which implies that they are mainly processed for consumption (91.7%). When probed further, the marketers claimed that they share their products to friends and relatives if unsold. The marketers sourced their products mainly from the wild (68.3%) and encountered several constrains. Approximately 51.7% faced seasonality of harvest and sale. An organized market will encourage market structure and good pricing per EI.

**Table 3:** Management practices of EIs in the study area.

Variables	Frequency	Percentage (%)
Types of EIs		
Termites (AKUEBE)	21	35.0
Palm weevil (UTUKURU)	18	30.0
Palm grub (ERU)	15	25.0
Others (OLOLO)	6	10.0
Means of storage		
Drums and pepper	27	45.0
Refrigerator	9	15.0
None	24	40.0
EI usage		
Consumption	55	91.7
Medicinal	5	8.3
Others	0	0.0
Supply		
Wild	41	68.3
Insect farmers	5	8.3
Wild and farmers	4	6.7
Constrains		
Irregular supply		
Seasonality of harvest	31	51.7
Storage facilities	9	15.0
Lack of finance	7	11.7
Lack of patronage	5	8.3
Transportation	8	13.3

### Mean qualities in kilograms of EIs sold in the markets

**Figure 1:**
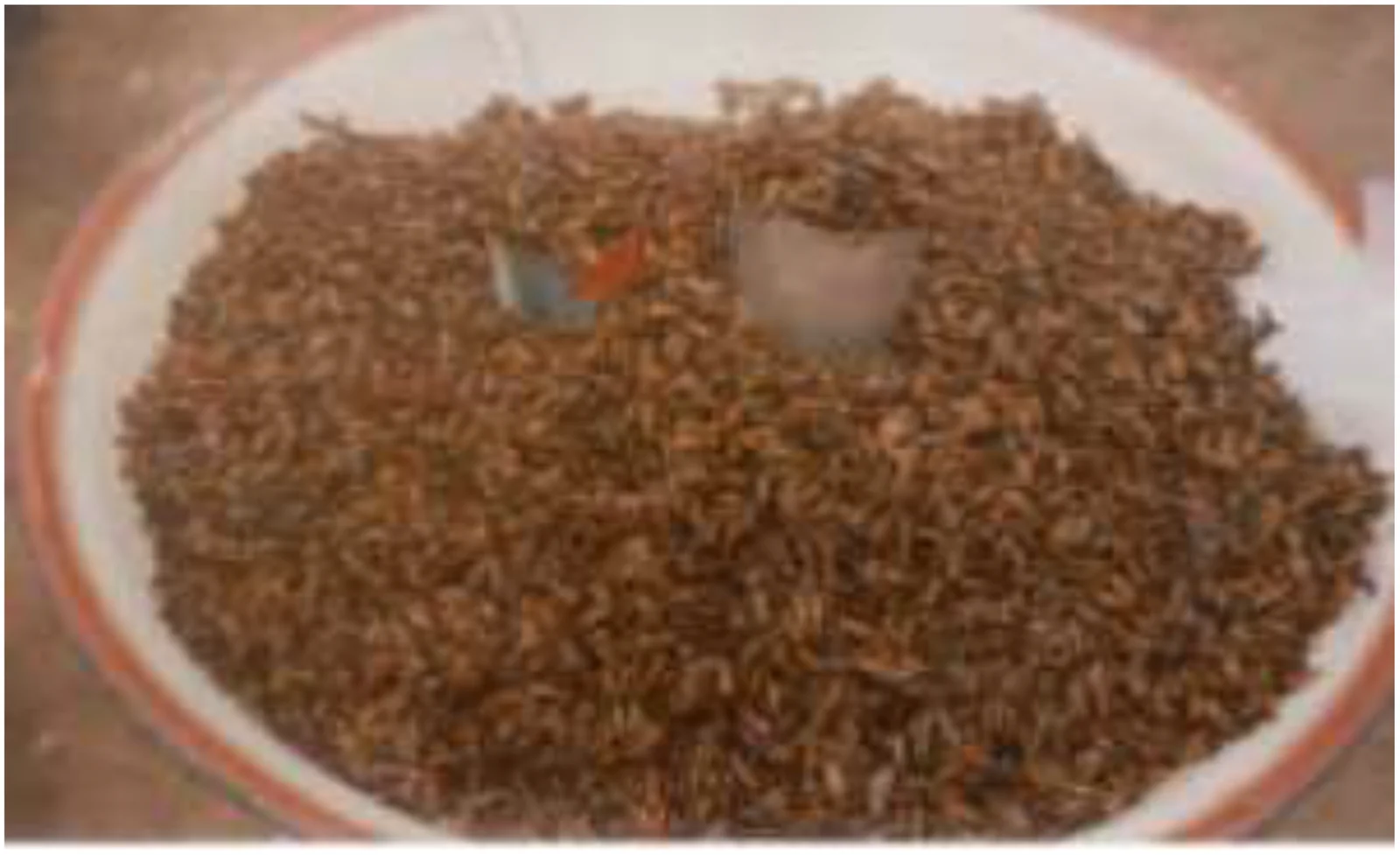
*Macrtotermes* spp (termites) on sale at Orie Ogbaku, Mbaitolu Imo State.

**Figure 2:**
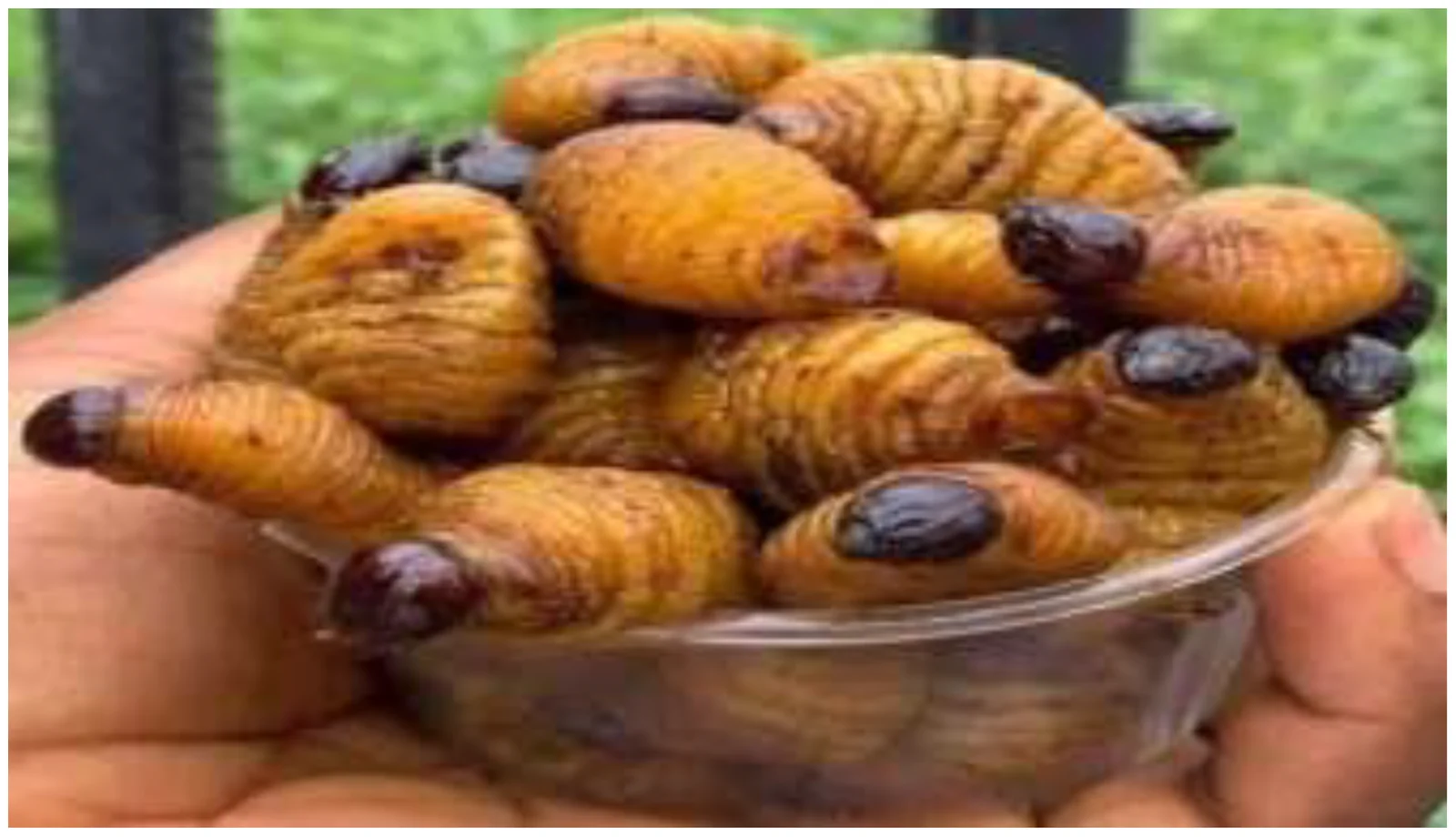
*Gonimbrasia bIelina* (Palm grub) on sale Eke Ukwu Owerri, Owerri Municipal Imo State.

**Figure 3:**
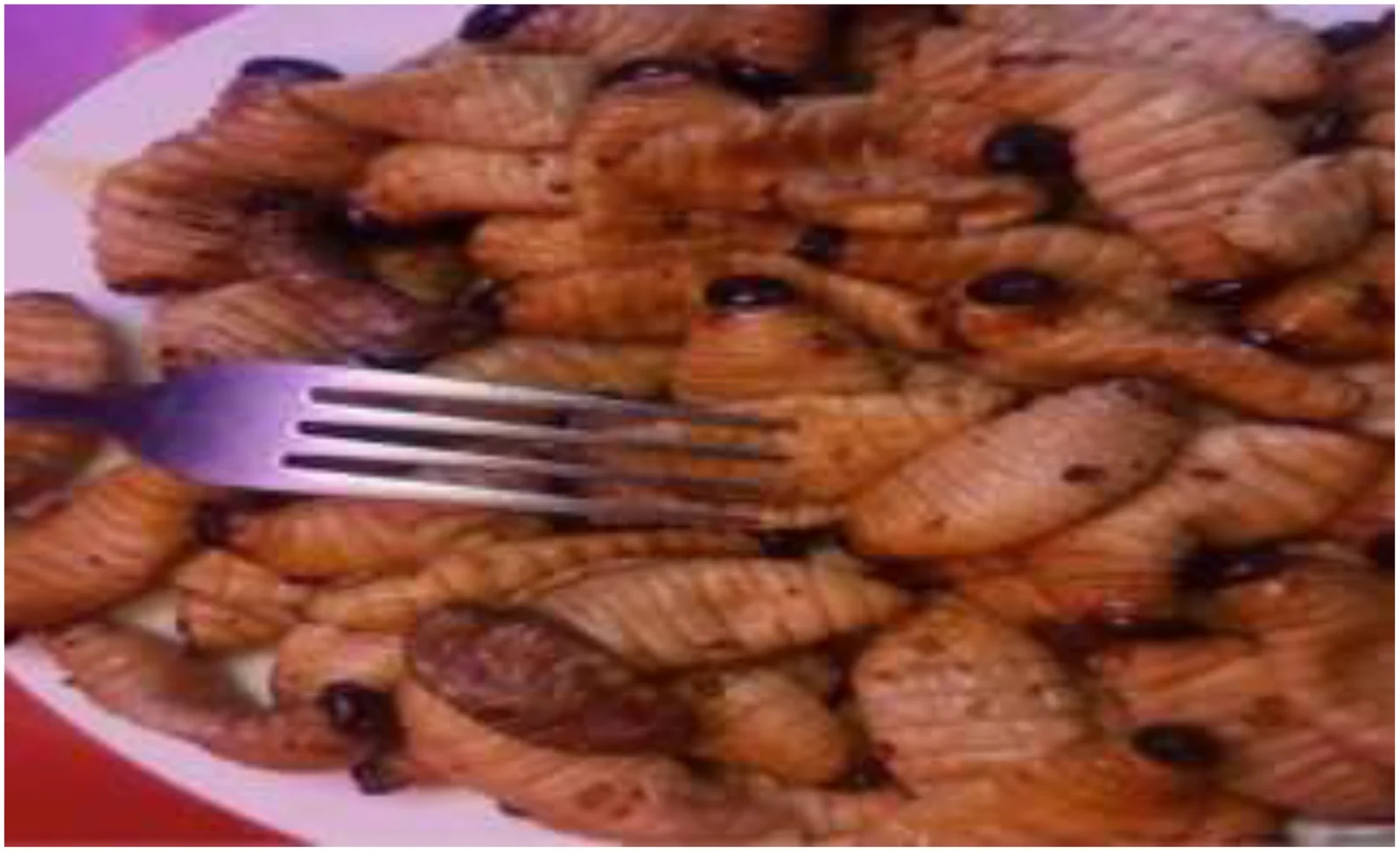
*Gomimbrasia belina* (Palm grub) on sale Eke Avutu Obowo, Imo State.

**Figure 4:**
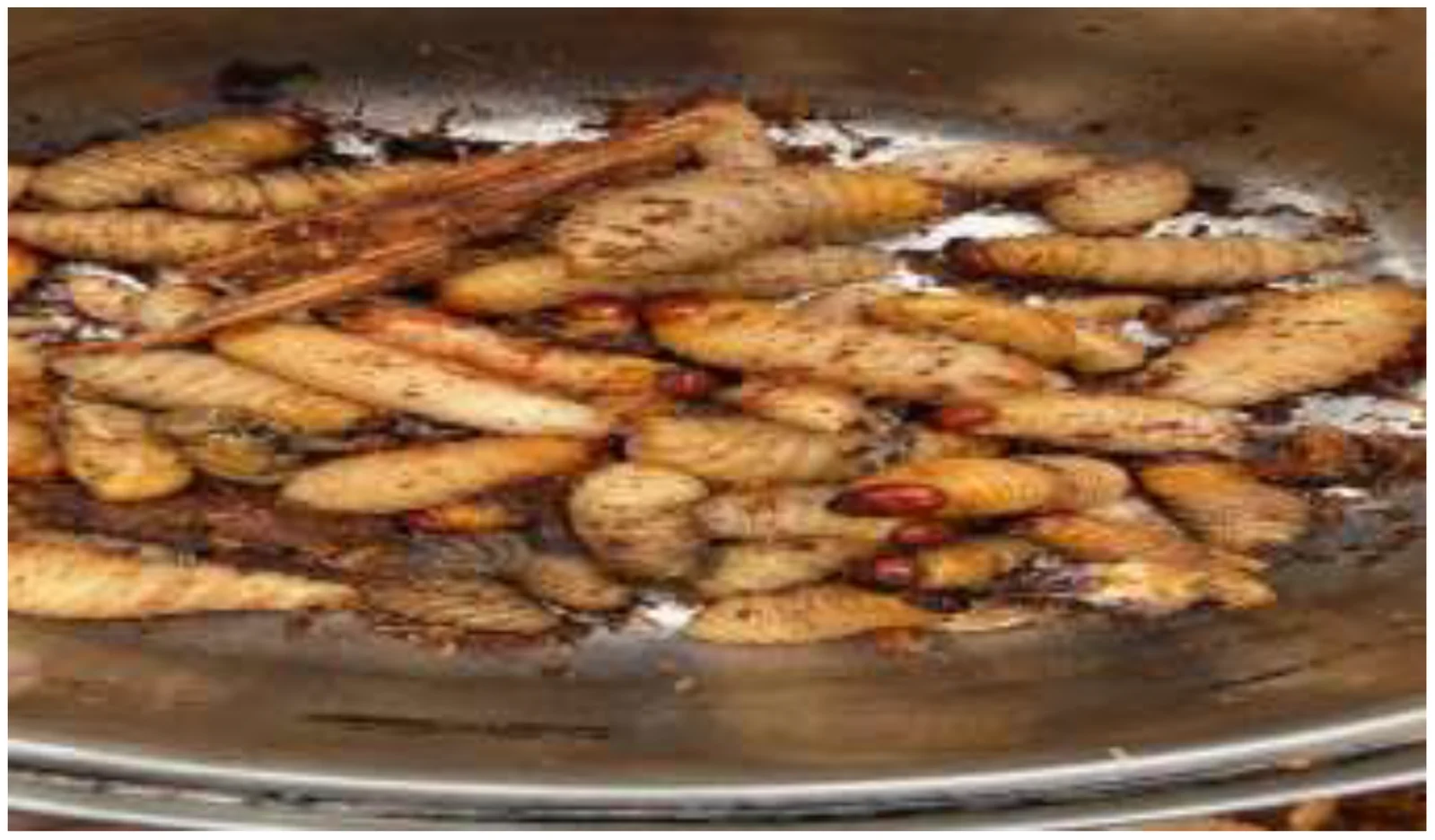
*Gonimbrasia belina* (Palm grub) on sale Eke Eziachi Orlu, Imo State.

**Figure 5:**
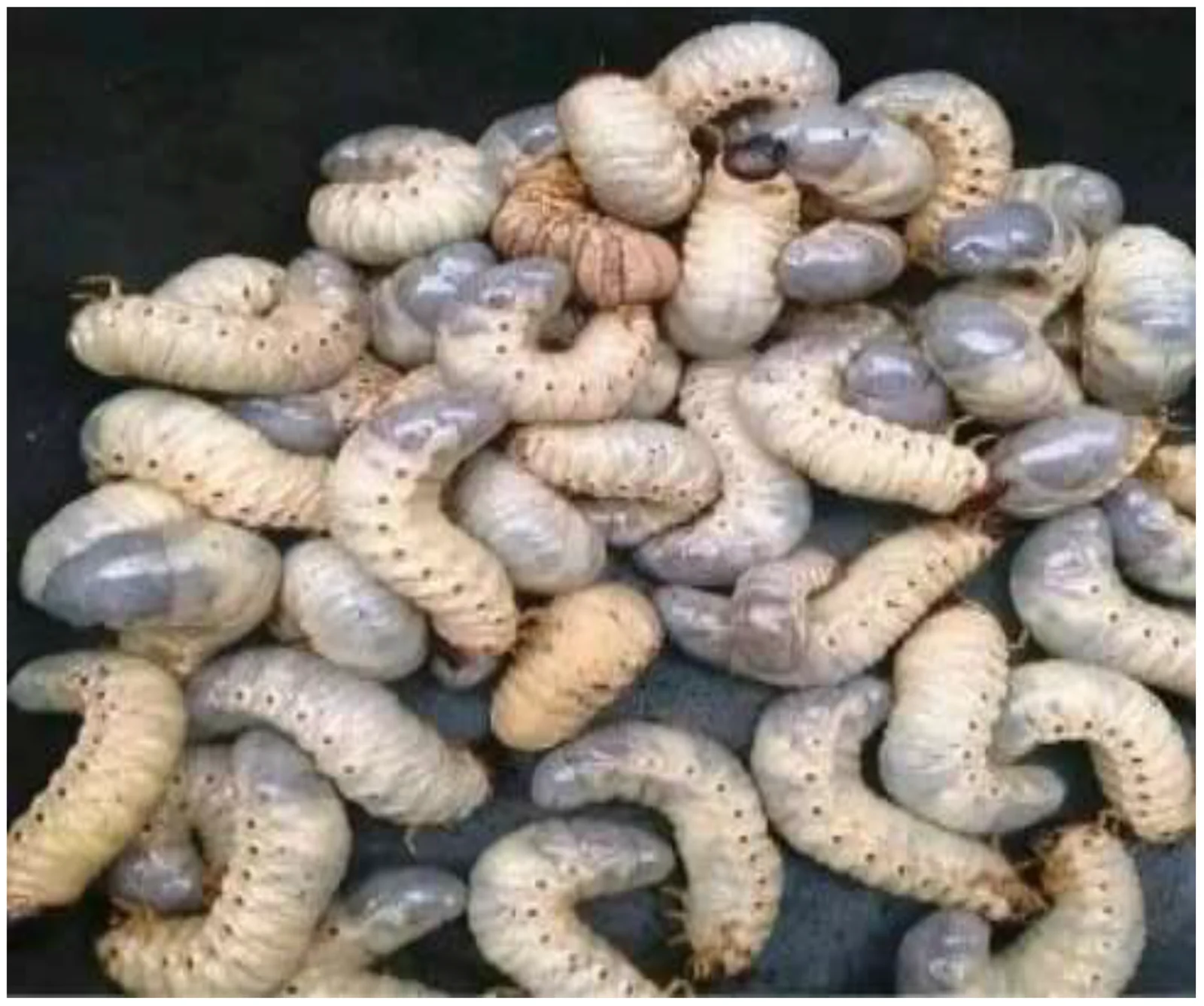
*Rhynchophorus* species (palm weevil) on sale at Orie Alaike Obowo Imo State.

**Figure 6:**
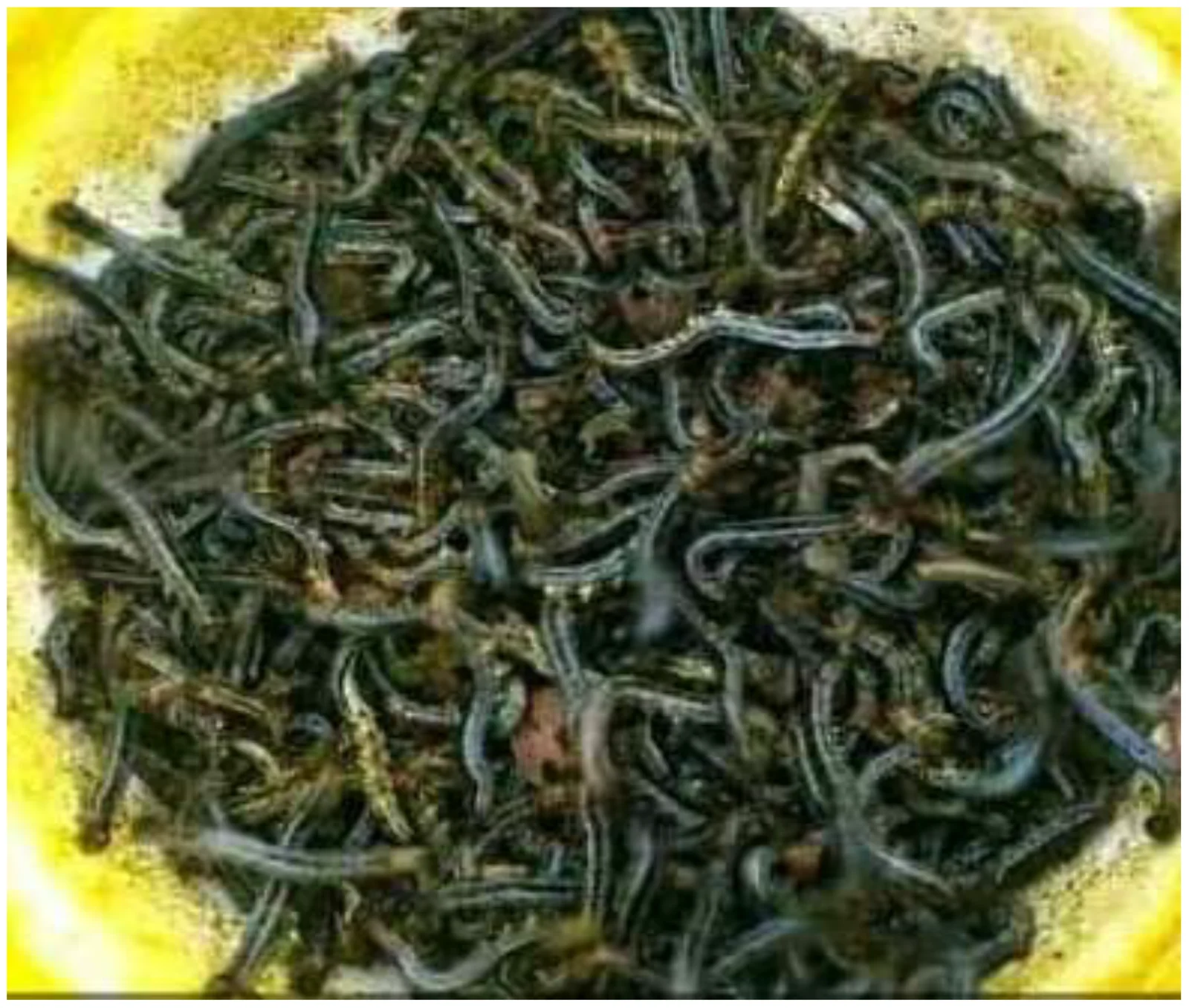
*Rhynchophorus* species (palm weevil) on sale at Orie Alaike Obowo Imo State.

Four species were offered for sale within the study area (**Table 4**). These were *Macrotermes* species, *Gonimbrasia belina*, *Rhynchophorus* species, and *Anaphae* species (**Figures 1**–**6**). *Macrotermes s*pecies had the highest significant difference in mean quality per marketer among the species. EIs were sold using spoons, milk and tomato tin cups, local mudu, or just placed in heaps with prices depending on the unit of measure, the species, and the market in question. For example, a mudu of OLOLO and termites that weighed 0.7 kg was sold for N400.00 while a tomato tin (0.1 kg) was sold for N100.00 in Orie Ogbaku in Mbaitolu and Eke Ukwu in Owerri Municipal. The same products were sold for N350.00 and N150.00, respectively, in Orie Okporo and Orie Alaike in Orlu and Obowo. In many rural markets, there was no fixed unit of measurement; EIs were sold in various containers with the prices depending on the quantity. In some cases, EIs were processed and pinned on sticks for sale in major roads/streets and bus stops in Owerri Municipal.

**Table 4:** Mean quantities (in kilograms) of EIs sold in the markets.

Species	Common names	Mean quantity (kg) per market
*Macrotermis belicosus*	AKU/AKUEBE	17 ± 0.2a
*Gonimbrasia belina*	UTUKURU	0.3 ± 0.2b
*Rhynchophorus species*	ERU	0.2 ± 0.3b
*Anaphae species*	OLOLO	0.4 ± 0.3b

Means with the same superscripts are not significantly different (*p* > 0.05).

## Conclusion and recommendation

EIs are marketed in Imo State with termites, African palm weevils and palm grubs, and OLOLO as the most marketed. In Nigeria, insects have been relatively neglected as a source of food. The results herein are an opportune time to start paying attention to these neglected/undervalued creatures—insects. The government should promote research and development leading to better utilization of insects. Trust must be given to the entrepreneurial potential of EI farming in terms of mass production and training on modern techniques. The government is advised to legislate on urbanization, bush burning, and illegal felling of trees to preserve the habitats of EIs.

## Data Availability

The raw data supporting the conclusions of this article will be made available by the authors, without undue reservation.
